# SEDT2/METTL14-mediated m6A methylation awakening contributes to hypoxia-induced pulmonary arterial hypertension in mice

**DOI:** 10.18632/aging.202616

**Published:** 2021-02-26

**Authors:** Xue-Liang Zhou, Feng-Jian Huang, Yang Li, Huang Huang, Qi-Cai Wu

**Affiliations:** 1 Department of Cardiac Surgery, The First Affiliated Hospital, Nanchang University, Nanchang 330006, Jiangxi, China

**Keywords:** pulmonary arterial hypertension, SETD2, METTL14, N6-methyladenine (m6A)

## Abstract

Pulmonary arterial hypertension (PAH) is a fatal disease whose molecular mechanism is unknown. The trimethylation of lysine 36 on histone 3 (H3K36me3) catalyzed by SETD2 and the modification of N6-methyladenine (m^6^A) mRNA mediated by METTL14 play important roles in a variety of normal and pathological biological processes. However, the role of these epigenetic controls in the pathogenesis of PAH remains unclear. In this study, the expression of SETD2 and METTL14 was elevated in pulmonary artery smooth muscle cells (PASMCs) of hypoxia-induced PAH mice. We further constructed a mouse model with SETD2 specific knockout in smooth muscle cells (SETD2^SM22α Cre^). Our results suggest that the lack of SETD2 in SMCs protected mice from hypoxia-induced PAH and significantly reduced right ventricular systolic pressure (RVSP), right ventricular/left ventricular plus septum [RV/(LV+S)] weight ratio, and pulmonary median width. In addition, the absence of SETD2 in SMCs alleviates the level of METTL14 expression and the m^6^A RNA methylation level in PAH SMCs. These results obtained from mice suggest that strategies that target the inhibition of SETD2/METTL14 activity may be a viable treatment for PAH in a clinical setting.

## INTRODUCTION

Pulmonary artery hypertension (PAH) is a life-threatening disease characterized by progressive pulmonary vascular remodeling, resulting in a sustained increase in Pulmonary artery pressure and eventually right heart failure [1–3]. The main histopathological features of PAH are vascular wall remodeling, which includes intimal hyperplasia, inner and outer membrane hypertrophy and extracellular matrix deposition. PAH involves complex structural and functional changes [4, 5]. Multiple cell types and growth factors are involved in the development of PAH. Sustained pulmonary vasoconstriction and vascular remodeling, characterized by concentric wall thickening and lumen occlusion of the small and medium pulmonary arteries (PAs), has convincing evidence that pulmonary smooth muscle cells (PASMCs) proliferation is a major feature of pulmonary artery remodeling and is the basis for the development of PAH [6, 7]. The constitutive proliferative phenotype of PASMCs is considered to be a key characteristic of PAH patients or animals that induce PAH [4, 8–10]. Thus, although current treatment for PAH can improve clinical symptoms, the progression of the disease is inevitable in most patients, and the mortality rate remains unacceptably high. The pathogenesis of PAH is complex and has not been fully elucidated. Therefore, a better understanding of the pathogenesis of the disease is crucial to identify new therapeutic targets.

Epigenetic regulation plays a key role in the evolution of PAH [11]. Among these factors, histone methyltransferases (HMTs) are tempting targets for disease intervention [12–14] because they are often maladjusted in the development of PAH and their enzyme activity can be used for treatment [15]. In mammalian cells, SETD2 is the main enzyme that catalyzes the trimethylation of lysine 36 on histone 3 (H3K36me3), a histone marker associated with an active transcriptional region [16]. Inactivation of SETD2 in the germ line of mice leads to defects in vascular remodeling, indicating that SETD2 plays a non-redundant role in development [17]. Cell culture-based studies have shown that there are defects such as chromosome separation, DNA repair and nucleotide pool reduction in SETD2 - deficient cells. Mechanism characteristics indicate that SETD2 mediated regulation of H3K36me3 and recruitment of DNMT3b ensure the fidelity of gene transcription initiation in embryonic stem cells [18]. In addition, SETD2 and H3K36me3 are involved in RNA splicing during gene transcription. SETD2 mediated H3K36me3 affects splicing site selection by recruiting morf-related gene 15 (MRG15) and polypyrimidine bundle binding protein 1 (PTB) [19]. However, in many cases, it is not clear whether SETD2 mediated changes in H3K36me3 are the driving force for PAH.

N6-methyl adenine (m^6^A), the most common internal modification of mRNA, is an important post-transcriptional gene regulation mechanism and plays an important role in various normal and pathological biological processes [20–23]. M^6^A modification is reversible and is added by methyltransferase complex and removed by m^6^A demethylase. M^6^A methyltransferase complex is composed of METTL3, METTL4, METTL14, WTAP, VIRMA, etc [24]. In contrast, FTO and AlkBH5 induced m^6^A demethylase. The effect of m^6^A on RNA stability depends on the m^6^A reader, including YTHDF1, YTHDF2, YTHDF3, and YTHDC1 [21, 22]. M^6^A modification was reported to be enriched near the H3K36me3 peak, and decreased in the overall range when the cell H3K36me3 was depleted [25]. Mechanistically, H3K36me3 is recognized as a key component of direct binding by METTL14 to the m^6^A methyltransferase complex (MTC), which promotes binding to m^6^A MTC adjacent to RNA polymerase II, thereby facilitating transcription of target genes [25]. However, the role of METTL14-mediated m^6^A modification in PAH is completely unknown.

Here, we constructed a mouse model with SETD2 specific knockout of smooth muscle cells (SETD2^SM22α-cre^). Our results suggest that the lack of SETD2 in SMCs protected mice from hypoxia-induced PAH and significantly reduced RVSP, [RV/(LV+S)], and pulmonary median width. Moreover, the deletion of SETD2 in SMCs significantly reduced the level of METTL14 and the methylation of m^6^A RNA induced by PAH. These results suggest that strategies that target the inhibition of SETD2/METTL14 activity may be a viable treatment for PAH in a clinical setting.

## RESULTS

### SMCs specific SETD2 deficient ameliorates pulmonary pressure in a hypoxia-induced mouse model of PAH

**Figure 1:**
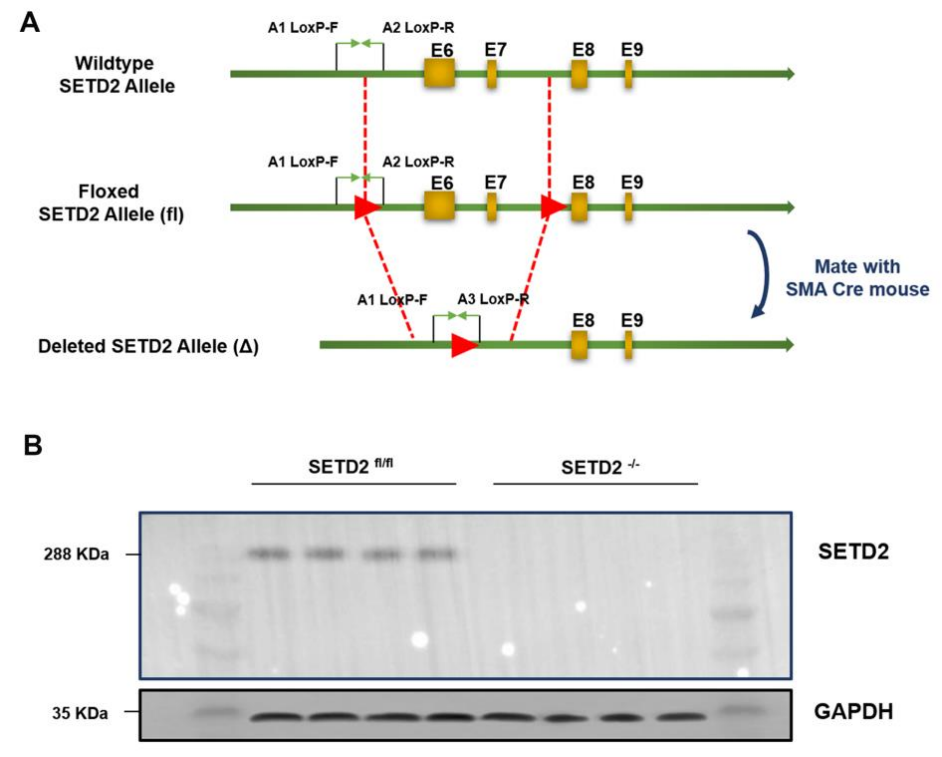
**Generation and characterization of mice with smooth muscle cells specific knockout of SETD2.** (**A**) The targeting strategy of mice with specific knockout of SETD2 in smooth muscle cells. (**B**) Western blotting was used to confirm the expression of SETD2 in smooth muscle cells from normal mice and SMCs SETD2 specific knockout mice.

**Figure 2:**
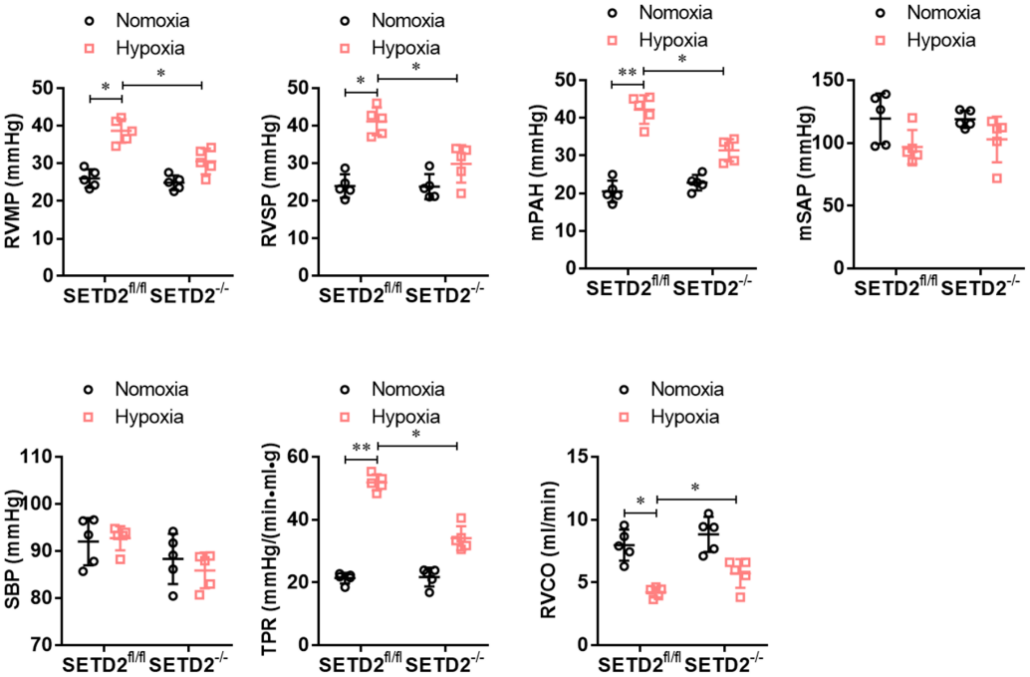
**SMCs specific SETD2 deficient ameliorates pulmonary pressure in a hypoxia-induced mouse model of PAH.** The hemodynamic measurements were performed to measure the right ventricular mean pressure (RVMP), right ventricular systolic pressure (RVSP) and mean pulmonary arterial pressure (mPAP). The left carotid artery cannulation was performed for systemic arterial pressure (mSAP) and systolic body pressure (SBP) measurement. Total pulmonary resistance (TPR) was calculated by the ratio of mPAP/RVCO. All the calculations were done with a LabChart8 (AD Instruments). All data were presented as Mean±SD (n=5). *P<0.05, **P<0.01 vs. corresponding group.

To explore the role of SETD2 in the vascular remodeling during PAH, we constructed a C57BL/6 mouse with specific knockout of SETD2 in SMCs (SETD2^SM22a-Cre^ C57BL/6 mice) (Figure 1A). The deleted expression of SETD2 in SMCs was confirmed by Western blotting (Figure 1B). Next, SETD2^SM22a-Cre^ mice and SETD2^fl/fl^ mice were subjected to induction of PAH under normobaric normoxic or hypoxic conditions (10% O_2_) for 28 days, respectively. After 28 days of hypoxic exposure, hypoxia significantly increased RVMP, RVSP, mPAP, TPR, and PAMT, compared with normoxic group, whereas RVCO in hypoxia treated groups was dramatically reduced (Figure 2). There were no significant differences in SBP and mSAP between hypoxic and normoxic groups (Figure 2). However, the mean of RVMP, RVSP, mPAP, TPR and PAMT were significantly lower in SETD2^SM22a-Cre^ mice than that in SETD2^fl/fl^ mice after hypoxic exposure (Figure 2). Furthermore, SETD2 deficiency in SMCs led to significant increases in RVCO in SETD2^SM22a-Cre^ mice compared with SETD2^fl/fl^ mice (Figure 2). Collectively, these data suggest that SETD2 deficiency in SMCs ameliorates PAH in hypoxia-induced mouse PAH model.

### SMCs specific SETD2 deficient ameliorates right ventricular function in a hypoxia-induced mouse model of PAH

**Figure 3:**
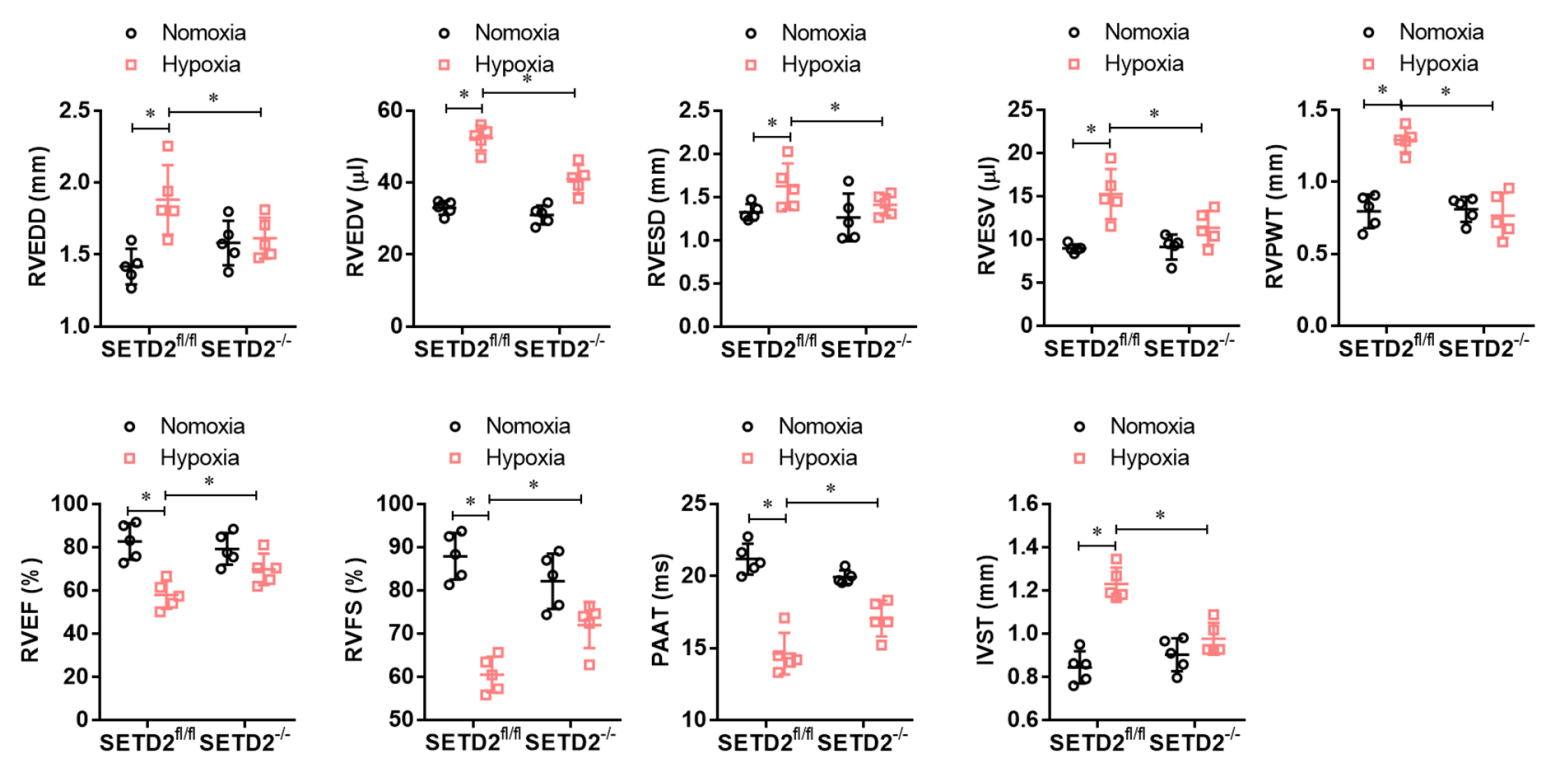
**SMCs specific SETD2 deficient ameliorates right ventricular function in a hypoxia-induced mouse model of PAH.** Echocardiography was used to measure the normalized cardiac cycle length measuring pulmonary acceleration time (PAAT/CL), RV end diastolic dimension (RVEDD), RV diastolic contraction size (RVESD), RV end diastolic volume (RVEDV), RV diastolic volume contraction (RVESV), RV cardiac output (RVCO), RV wall thickness (RVWT), the thickness of interventricular septum (IVS), RV ejection fraction (RVEF) and RV shortening fraction (RVFS). All data were presented as Mean**±**SD (n=5). *P<0.05, **P<0.01 vs. corresponding group**.**

To explore whether SMCs specific SETD2 deficient ameliorates right ventricular dysfunction in mouse PAH model, we performed echocardiography. As shown in Figure 3, hypoxia significantly increased RVEDD, RVEDV, RVESD, RVESV, RVPWT and IVST and significantly decreased RVEF, RVFS and PAAT/CL compared with normoxic group. Consistently, SMCs specific SETD2 deficient obviously reduced RVEDD, RVEDV, RVESD, RVESV, RVPWT and IVST, and enhanced RVEF, RVFS and PAAT/CL compared with SETD2^fl/fl^ mice in hypoxia group. These data indicate that hypoxia-induced PAH mice develop significant right ventricular dysfunction, which could be ameliorated by SMCs specific SETD2 deficient.

### SMCs specific SETD2 deficient attenuates pathological remodeling of pulmonary artery and right ventricular hypertrophy

**Figure 4:**
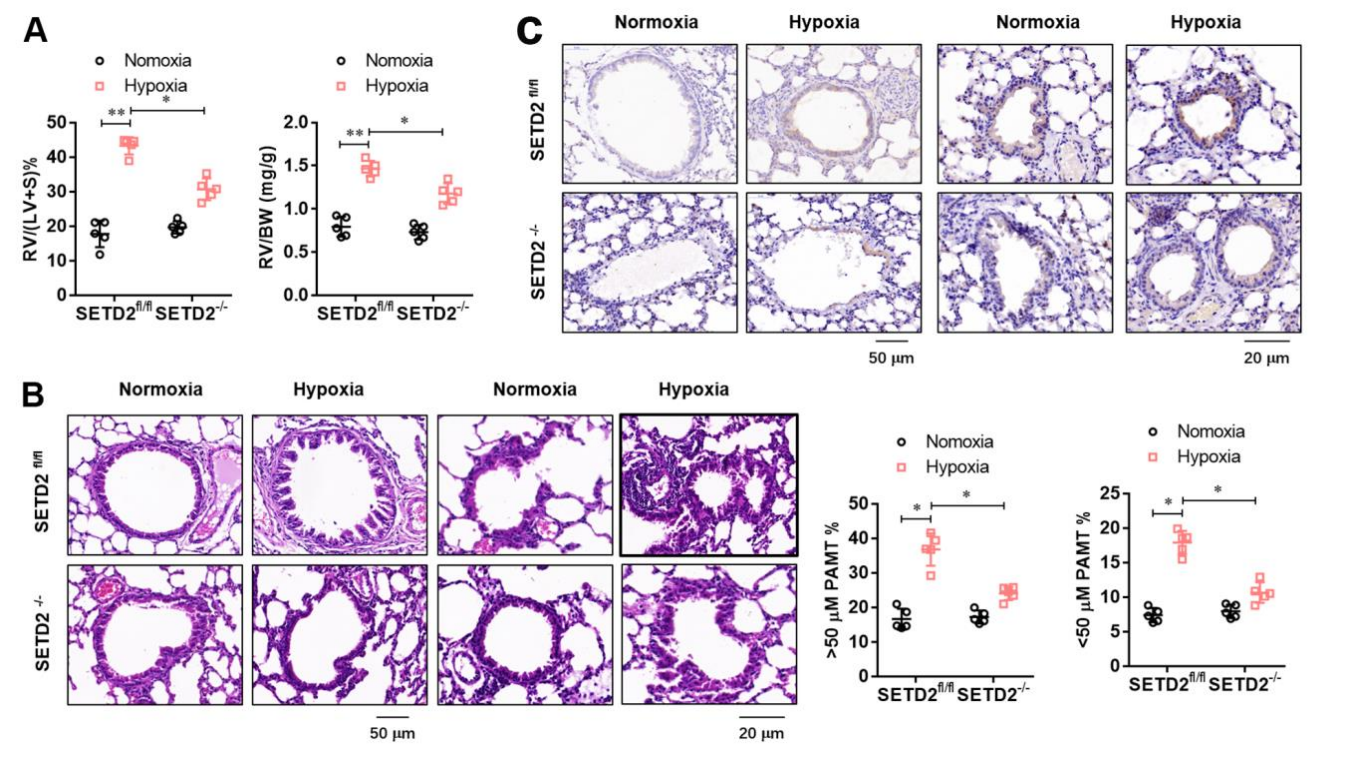
**SMCs specific SETD2 deficient attenuates pathological remodeling of pulmonary artery and right ventricular hypertrophy.** (**A**) The right ventricular hypertrophy was evaluated by the RV/BW ratio (the ratio of the right ventricular mass to the sum of body masses) and RV/(L+S) ratio (the ratio of the right ventricular mass to the sum of the left ventricular and septal masses). (**B**) Haematoxylin and eosin (HE) staining and the analyzed PAMT ration of both small and medium pulmonary arteries were used to evaluate the pulmonary blood vessel remodeling during the course of PAH development. (**C**) Histochemical staining of SETD2 immunostaining was used to detect the SETD2 expression in lung blood vessels. All data were presented as Mean±SD (n=5). *P<0.05, **P<0.01 vs. corresponding group.

To further evaluate the effect of SMCs specific SETD2 deficient on pathological remodeling of pulmonary artery and right ventricular hypertrophy, we firstly evaluated the RV/BW ratio (the ratio of the right ventricular mass to the sum of body masses) and RV/(L+S) ratio (the ratio of the right ventricular mass to the sum of the left ventricular and septal masses), a marker of hypertrophy in the right ventricle due to elevated right ventricular pressure and afterload. As shown in Figure 4A, RV/BW and RV/(LV+S) in hypoxia treated mice significantly increased compared with normoxic mice, but SMCs specific SETD2 deficient significantly decreased RV/BW and RV/(LV+S). Histological studies were next conducted to demonstrate the effect of SETD2 on pulmonary blood vessel remodeling during the course of PAH development. Remarkably, staining with haematoxylin and eosin (HE) (Figure 4B) demonstrated that loss of SMCs SETD2 significantly attenuated the PAMT ration of both small and medium pulmonary arteries, indicating the alleviated blood vessel remodeling (Figure 4B). Histochemical staining of SETD2 immunostaining revealed that the SETD2 expression in blood vessels of SETD2^fl/fl^ mice were profoundly enhanced in response to chronic hypoxic exposure. In sharp contrast, the pulmonary arteries originated from SETD2^SM22a-Cre^ mice were remodeled to a significantly less degree and harbored much lower SETD2 expressions (Figure 4C). These data indicate that SMCs specific SETD2 deficient obviously reverses pathological remodeling of pulmonary artery and right ventricular hypertrophy in PAH mice model induced by hypoxia.

### SETD2 mediated H3K36me3 and METTL14 mediated m^6^A RNA modification are involved in hypoxia-induced PAH

**Figure 5:**
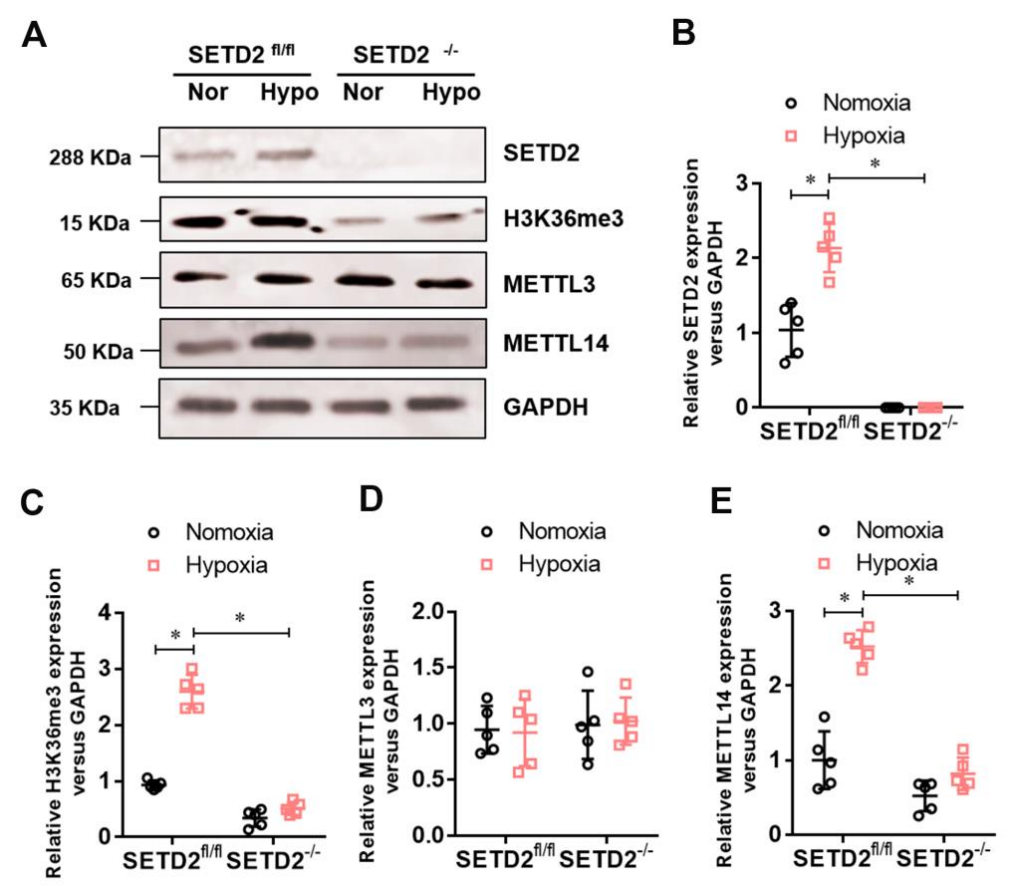
**The expression of SETD2 mediates H3K36me3 and METTL14 are enhanced in hypoxia-induced PAH, whereas impaired by SMCs specific SETD2 deficient.** (**A**) Represent images of Western blotting analysis. The statistical data showed that hypoxia markedly elevated the protein level of SETD2 (**B**), H3K36me3 (**C**) and METTL14 (**E**) in SMCs, but SMCs specific SETD2 deficient impaired the level of the protein level of H3K36me3 and METTL14. However, the protein level of METTL3 (**D**) was changed neither by hypoxia nor SMCs specific SETD2 deficient. All data were presented as Mean±SD (n=5). *P<0.05 vs. corresponding group.

**Figure 6:**
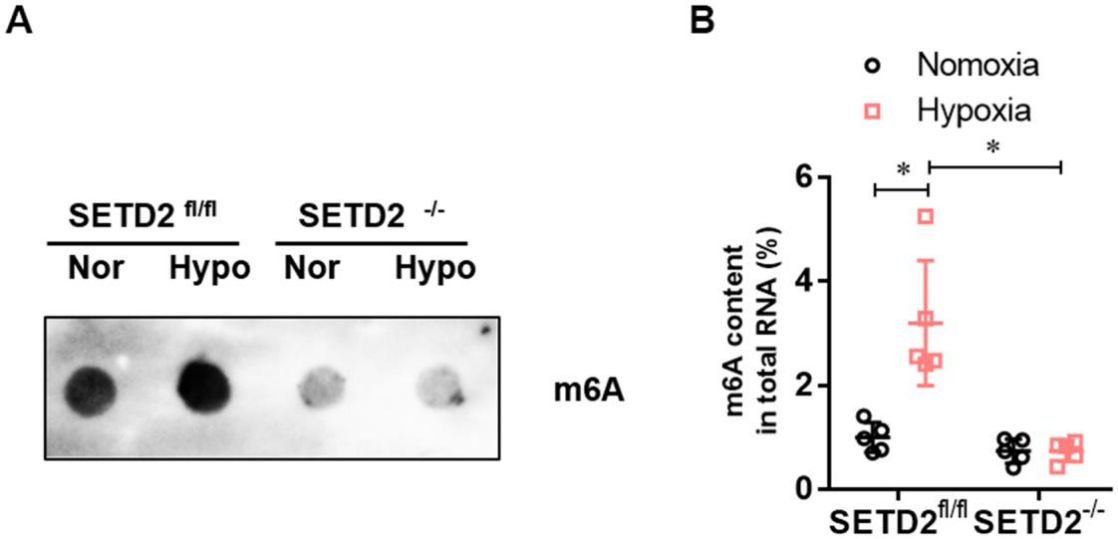
**The level of m**^6^**A RNA modification is elevated in hypoxia-induced PAH, whereas impaired by SMCs specific SETD2 deficient.** (**A**) The content of m^6^A was quantitatively determined by colorimetry. (**B**) The content of m^6^A was qualitatively evaluated by Dot blot. All data were presented as Mean±SD (n=5). *P<0.05 vs. corresponding group.

To investigate whether SETD2 mediates H3K36me3 is involved in PAH, we evaluated the protein level of SETD2 and H3K36me3. Western blot analysis showed that hypoxia markedly elevated the protein level of SETD2 and H3K36me3 in SMCs (Figure 5A), but SMCs specific SETD2 deficient impaired the level of the protein level of SETD2 (Figure 5A, 5B) and H3K36me3 (Figure 5A, 5C). These data suggest that SETD2 mediates H3K36me3 is involved in PAH. As H3K36me3 is recognized and bound directly by METTL3/14 and deliver the m^6^A modification. We then evaluated the protein level of METTL3, METTL14 and the m^6^A modificated RNA level. Western blot analysis also showed that hypoxia markedly elevated the protein level of METTL14 in SMCs (Figure 5A, 5E), but SMCs specific SETD2 deficient impaired the level of the protein level of METTL14 (Figure 5A, 5E). However, the protein level of METTL3 was changed neither by hypoxia nor SMCs specific SETD2 deficient (Figure 5A, 5D). Moreover, the total m^6^A modificated RNA level, indicated by dot blot (Figure 6A) and m^6^A RNA methylation quantification (Figure 6B), were significantly enhanced in hypoxia induced PAH group, whereas diminished by SMCs specific SETD2 deficient. Collectively, these results demonstrated that SETD2 mediates H3K36me3 and METTL14 mediated m^6^A RNA modification contribute to hypoxia-induced pulmonary arterial hypertension in mice.

## DISCUSSION

Epigenetic changes caused by hypoxia can lead to permanent changes in early fetal development, increasing the risk of PAH in adults [11]. Unlike genetic mutations, epigenetic changes are pharmacologically reversible, making them an attractive target for the treatment of PAH. Data from this study showed that SMCs-specific SETD2 deletion resulted in the inactivation of H3K36me3 modification, which reversed hypoxia-induced PAH. Chronic hypoxia exposure can lead to significant increase in RVSP, increase in RV/(LV+S), and significant thickening of the middle membrane of pulmonary arterioles. However, the specific knockout of SETD2 in SMCs inhibits these increases. Furthermore, we showed that METTL14 mediated m^6^A RNA modification was elevated in the SMCs from hypoxia-induced PAH mice, which was similarly diminished by specific knockout of SETD2 in SMC. This novel information partially clarified that SETD2 mediates H3K36me3 and METTL14 mediated m^6^A RNA modification contribute to hypoxia-induced pulmonary arterial hypertension in mice.

In the development of PAH, the Warburg effect (glycolytic shift) and mitochondrial fission are critical factors that determine the phenotypic characteristics of the disease, such as proliferation, apoptotic resistance, migration, endothelial-mesenchymal transition, and extracellular matrix stiffness [3–5, 7]. Current therapies have focused on vasodilation and antithrombotic protection, but have not restored these abnormal phenotypes, suggesting the need to target other pathways. The multi-factor characteristics of PAH suggest that epigenetic changes are potential determinants of vascular remodeling [26, 27]. In recent years, studies on molecular markers for epigenetic changes in PAH have steadily increased [28, 29]. These experimental and clinical studies have shown that epigenetic processes, such as DNA methylation, ubiquitination, miRNA-dependent gene regulation, and HDACs are associated with PAH [26, 30]. Histone methylation is a process of transcriptional promotion or inhibition of post-translational modification based on specific methylated residues (lysine or arginine) and the number of methyl groups added [31]. However, histone methylation involved in PAH has been rarely reported. Our previous studies showed that NSD2, catalytic dimethylation of H3K36 (H3K36me2) changes in energy metabolism are involved in monocrotaline-induced PAH [32]. On the other hand, the SETD2^fl/fl^ and SETD2^-/-^ groups showed very similar pulmonary pressure and right ventricular function, which suggest that SETD2 mediated H3K36me3 may not be critical under normal physiological conditions. Moreover, SMCs specific SETD2 deficient did not alert the level of m^6^A and METTL14 under normal physiological conditions, whereas impaired the PAH induced elevation of m6A and METTL14. Hence, we think that METTL14 protein and m6A RNA are pathologically involved in the progression of PAH. Thus, this study further demonstrated that SETD2 mediated H3K36me3 is pathologically involved in hypoxia-induced PAH in mice.

Muscularization of vascular wall and phenotypic changes of endothelial cells lead to vascular remodeling, which is the main pathological feature of PAH [33, 34]. Arterioles obstructed by phenotypic changes and proliferation of cross-differentiated pulmonary artery endothelial cells (PAECs) are considered irreversible [35]. However, there is also some evidence that pulmonary artery SMCs can be transferred between proliferative and non-proliferative states, raising the possibility that this aspect of vascular reconstruction may be reversible [34, 36, 37]. The effect of m^6^A post-transcriptional modification on proliferation control has received extensive attention in recent years. METTL3, the main m^6^A methyltransferase, inhibits cell differentiation and apoptosis and promotes cell proliferation by increasing the translation of c-myc, bcl-2 and PTEN in leukemia [38]. WTAP, another member of m^6^A writer, enhances proliferation, migration, invasion, and tumorigenicity of glioblastoma cells in xenotransplantation by mediating the phosphorylation of epidermal growth factor receptor (EGFR) and AKT [39]. As far as we know, regulators of m^6^A play an important role in proliferation, especially in the development of cancer. In recent years, studies have found that total Panax notoginseng saponin regulate WTAP/p16 signal through m^6^A, and inhibit proliferation, migration and intimal hyperplasia of vascular smooth muscle cells [40]. However, the role of m^6^A post-transcriptional modification in the formation of PAH is unclear. In this study, we demonstrated that the total m^6^A modified RNA level was significantly increased in the hypoxia-induced PAH group, while the SMCs-specific SETD2 defect reduced the total m^6^A modified RNA level.

In summary, our study preliminarily found that SETD2 mediated H3K36me3 was involved in the occurrence and development of hypoxia-induced PAH through METTL14-mediated m^6^A RNA modification. Both SETD2 and METTL14 are promising targets for the prevention and treatment of PAH.

## MATERIALS AND METHODS

### Generation and characterization of mice

All animals were maintained in a specific pathogen-free facility. SM22α^Cre^ transgenic mice were purchased from Beijing Biocytogen Co. Ltd. (Beijing, China) SETD2^fl/fl^ C57BL/6 mice were generated by Shanghai Nanfang Research Center for Model Organisms, using conventional homologous recombination in embryonic stem cells, and the targeting strategy was shown in Figure 1A. All animal experiments were performed in accordance with the guidelines of NIH and under approved protocols of the Animal Care and Use Committee of Nanchang University.

### Chronic hypoxia induced PAH mice model

At the age of 10–12 weeks, male SETD2^SM22a-Cre^ mice and their WT littermates (SETD2^fl/fl^) were randomly assigned to the normoxia group (N) and hypoxia group (H). The normoxia group was exposed to room air, whereas the hypoxia-group mice were placed in a ventilated chamber with 10% O_2_ and CO_2_ < 0.5% environment. The environment was established by a mixture of room air and nitrogen with a detector (RCI Hudson, Anaheim, CA, USA) which monitored and controlled the fractional concentration of O_2_ automatically.

### Antibodies

Antibodies against SETD2, H3K36me3, METTL3 and METTL14 were purchased from Cell Signaling Technologies (Danvers, MA, USA), GAPDH and m^6^A antibodies were purchased from Abcam (Cambridge, MA, USA).

### Measurement of hemodynamics

After normal or hypoxic exposure, mice were anesthetized with sodium pentobarbital (60 mg/kg) and the hemodynamic measurements were performed as previously. In brief, the 25 g needle with a pressure sensor (FTH-1211B-0018, Primetech) were inserted through the jugular vein to measure the right ventricular mean pressure (RVMP), right ventricular systolic pressure (RVSP), mean pulmonary arterial pressure (mPAP), and the left carotid artery cannulation was performed for systemic arterial pressure (mSAP) and systolic body pressure (SBP) measurement. All the calculations were done with a LabChart8 (AD Instruments). Total pulmonary resistance (TPR) was calculated by the ratio of mPAP/RVCO.

### Echocardiography

Echocardiography was measured using the Vevo 2100 high-resolution imaging system (Visual Sonics Inc.) as previously [32]. Normalized cardiac cycle length measuring pulmonary acceleration time (PAAT/CL), RV end diastolic dimension (RVEDD), RV diastolic contraction size (RVESD), RV end diastolic volume (RVEDV), RV diastolic volume contraction (RVESV), RV cardiac output (RVCO), RV wall thickness (RVWT), the thickness of interventricular septum (IVS), RV ejection fraction (RVEF) and RV shortening fraction (RVFS). All echocardiographic studies were analyzed by genotypic blind method.

### Histological and immunohistochemical analysis

Histological and immunohistochemical analysis as described previously [32]. In brief, lung specimens were collected and stained with hematoxylin-eosin (HE). Immunohistochemical staining was performed on paraffin embedded equivalent slides (5 m) using a quick toolkit (Beyotime, Jiangsu, China) and stained with anti-SETD2 antibody (Abcam,1:1000 dilution).

### Western blotting analysis

Western Blotting analysis were performed as described previously [32]. Briefly, the protein was isolated by RIPA lysis buffer (Beyotime) and the protein concentration was determined using the BCA protein detection kit (Beyotime), then transferred to PVDF membranes (Millipore, inc., USA) by SDS-PAGE. The membranes were blocked with 5% non-fat dry milk in TBS-T (20 mM Tris, 137 mM NaCl, 0.05% Tween-20, pH 7.4) at room temperature for 3 h and then incubated with primary antibodies at 4° C.

### Dot blot

Total RNA was isolated by TriZol (Thermo Fisher) and loaded to an Amersham Hybond-N+ membrane (GE Healthcare, USA). After UV crosslinking and PBST washing, they were then stained with 0.02% methylene blue (Chinese worker) and the scans showed the total amount of incoming RNA. After incubation with 5% non-fat milk, the membrane was incubated overnight at 4° C with specific m^6^A antibody (1:1000, Millipore). After incubation with hrp-labeled anti-mouse immunoglobulin G (IgG) for 1 hour, the blobs were observed with an imaging system (Bio-Rad, USA).

### Total RNA m^6^A quantification

Total RNA was extracted using the TriZol kit (Thermo Fisher), and polyadenylate mRNA was purified using the GenElute™mRNA Miniprep kit (Sigma-Aldrich, Germany) according to the manufacturer's protocol. EpiQuik™ m^6^A RNA methylation quantitative kit (colorimetry)(Epigentek, USA) was then used to determine the total level of m^6^A in the treated cells. Briefly, 500 ng RNA was added to each pore, followed by capture and detection antibodies. After multiple incubation, the content of m^6^A was quantitatively determined by colorimetry at the wavelength of 450nm and calculated according to the standard curve.

### Statistical analysis

All data were expressed as mean ± standard deviation and analyzed by SPSS 11.0 statistical software. One-way ANOVA was used to compare the differences among the groups. *P*<0.05 was considered statistically significant.
